# Crystal structure of 2,3-dimeth­oxy-*N*-(4-nitro­phen­yl)benzamide

**DOI:** 10.1107/S2056989017017741

**Published:** 2018-01-01

**Authors:** Mavişe Yaman, Zainab M. Almarhoon, Şükriye Çakmak, Halil Kütük, Güngör Meral, Necmi Dege

**Affiliations:** a Ondokuz Mayıs University, Faculty of Arts and Sciences, Department of Physics, 55139 Samsun, Turkey; b Chemistry Department, Science Faculty, King Saud University, Riyadh, Saudi Arabia; c Sinop University, Environmental Health Programme, 57000 Sinop, Turkey; d Ondokuz Mayıs University, Faculty of Arts and Sciences, Department of Chemistry, 55139 Samsun, Turkey

**Keywords:** crystal structure, nitro­phen­yl, methyl­acetamide, benzamide, di­meth­oxy­benzene, Hirshfeld surface

## Abstract

In the title compound, C_15_H_14_N_2_O_5_, the benzene rings are nearly coplanar, making a dihedral angle of 4.89 (8)°. An intra­molecular N—H⋯O hydrogen bond occurs between the imino and meth­oxy groups. In the crystal, weak C—H⋯O hydrogen bonds link the mol­ecules into supra­molecular chains propagating along the *a-*axis direction. π–π stacking is observed between parallel benzene rings of neighbouring chains, the centroid-to-centroid distance being 3.6491 (10) Å. Three-dimensional Hirshfeld surface analyses and two-dimensional fingerprint plots have been used to analyse the inter­molecular inter­actions present in the crystal.

## Abstract 2

In the crystal, inter­molecular weak C—H⋯O hydrogen bonds link the mol­ecules into the supra­molecular chains propagating along the *a* axis.

## Chemical context   

Amides have a very important place in both organic and biological chemistry. They are used as building blocks for natural products such as proteins and peptides. However, amides are not restricted to biological systems, but also have a wide range of uses in pharmaceutical chemistry (Khalafi-Nezhad *et al.*, 2005 ▸; Valeur & Bradley, 2009 ▸). Many amide derivatives have been found to possess anti­tumor, anti­microbial, anti-HIV, anti-inflammatory, anti­convulsant, anti­bacterial, anti­fungal, analgesic and anti­cancer properties (Kushwaha *et al.*, 2011 ▸; Fu *et al.*, 2010 ▸; Carbonnelle *et al.*, 2005 ▸; Siddiqui *et al.*, 2008 ▸). Benzamides and their derivatives are compounds of biological and pharmaceutical importance. A variety of benzamide derivatives have been synthesized by the inter­action of aniline derivatives that carry electron-donating groups (anisidines, toluidines) and acyl chlorides (2,3-di­meth­oxy­benzoyl chloride and 3-acet­oxy-2-methyl­benzoyl chloride) in a slightly basic medium (Cakmak *et al.*, 2016 ▸; Demir *et al.*, 2015 ▸).
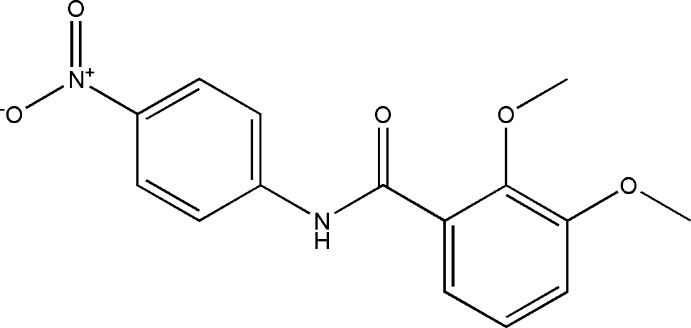


## Structural commentary   

**Figure 1:**
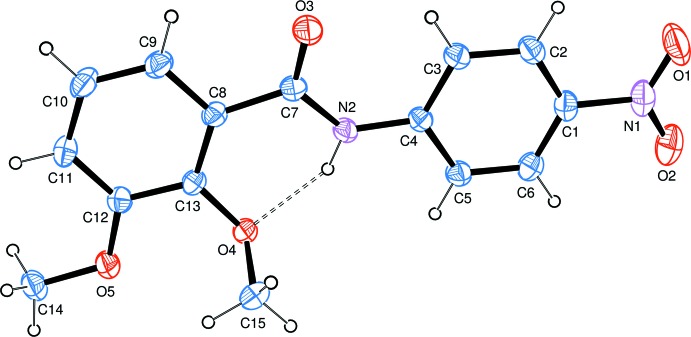
The mol­ecular structure of the title compound, with displacement ellipsoids drawn at the 50% probability level. The intramolecular N—HċO (Table 1 ▸) hydrogen bond is shown as a double dashed line.

The mol­ecular structure of the title compound is shown in Fig. 1 ▸. The bond distances and angles are found to be in good agreement with those in analogous structures (Demir *et al.*, 2015 ▸; Tahir *et al.*, 2011 ▸). In the mol­ecule, the benzene rings are nearly coplanar, with a dihedral angle of 4.89 (8)°. An intra­molecular N—H⋯O hydrogen bond (Table 1 ▸) occurs between the imino and meth­oxy groups.

## Supra­molecular features   

**Figure 2:**
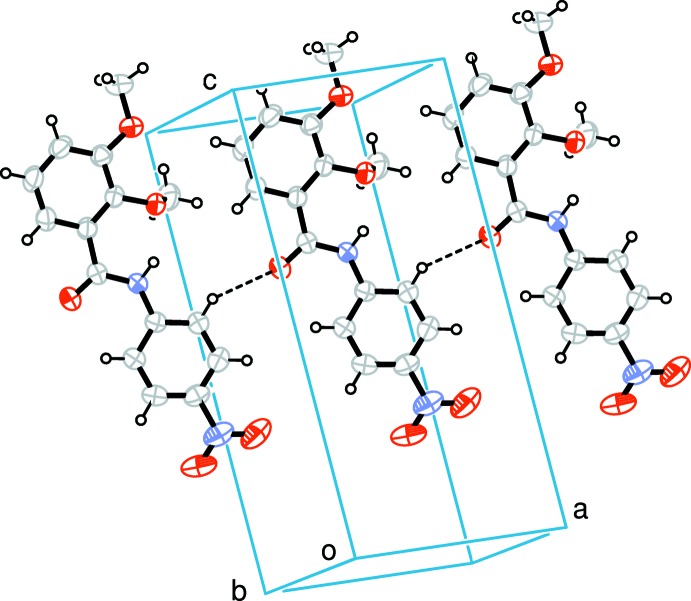
Packing of the title compound in the unit cell. Dashed lines indicate the C—H⋯O hydrogen bonds (see Table 1 ▸).

In the crystal, adjacent mol­ecules are linked by weak C—H⋯O hydrogen bonds, forming supra­molecular chains propagating along the *a*-axis direction (Table 1 ▸, Fig. 2 ▸). π–π stacking is observed between parallel benzene rings of adjacent chains, the centroid-to-centroid distance being 3.6491 (10) Å.

## Hirshfeld surface analysis   

Three-dimensional Hirshfeld surfaces (HS) were generated using *Crystal Explorer 3.1* (Wolff *et al.*, 2013 ▸) based on the results of the single crystal X-ray diffraction studies. Two-dimensional fingerprint plots (FPs) provide a visual representation of crystal-packing inter­actions in the structure. The HS is a useful tool for describing the surface characteristics and gaining additional insight into the inter­molecular inter­actions of the mol­ecules.

**Figure 3:**
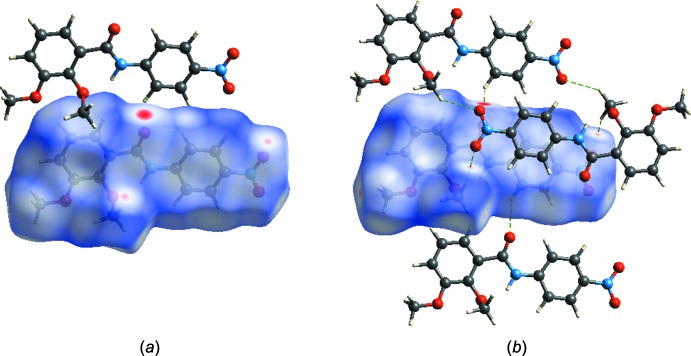
Hirshfeld *d*_norm_ (*a*) for 2,3-dimeth­oxy-*N*-(4-nitro­phen­yl)benzamide and (*b*) showing the hydrogen bonding.

**Figure 4:**
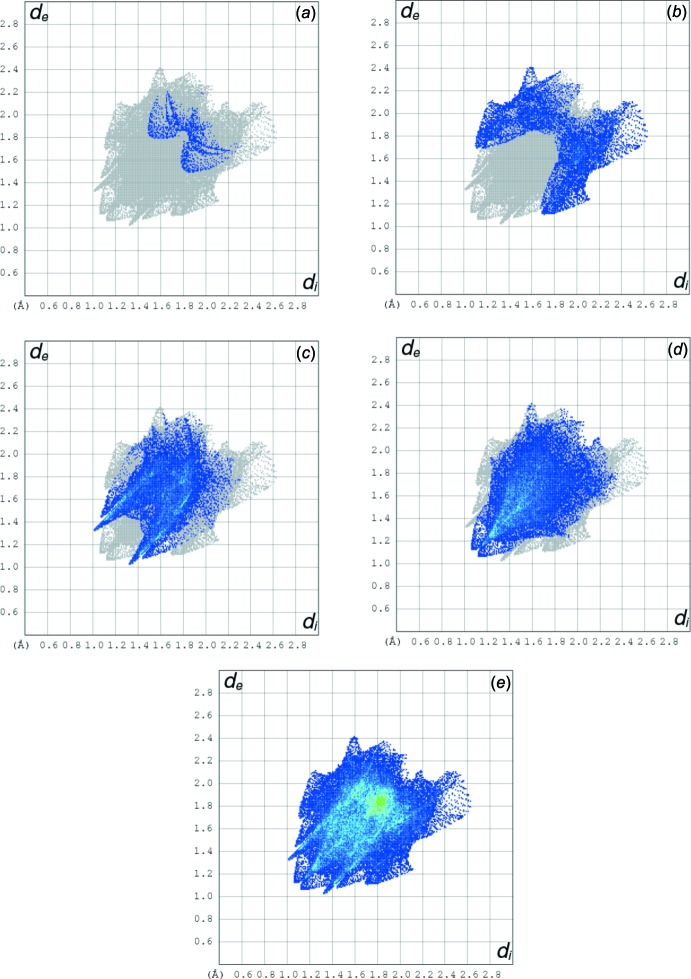
Hirshfeld surface fingerprint of the title compound, (*a*) N_inside_⋯H_outside_ (2.3%), (*b*) C_inside_⋯H_outside_ (15.7%), (*c*) O_inside_⋯H_outside_ (29.7%), (*d*) H_inside_⋯H_outside_ (38%), (*e*) all atoms_inside_⋯all atoms_outside_ (100% of total inter­actions).

**Figure 5:**
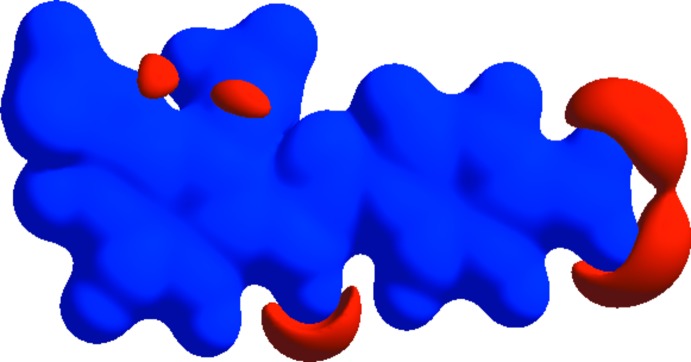
Electrostatic potential mapped on the Hirshfeld surface with ±0.25 au

The mol­ecular Hirshfeld surface, *d*_norm_, is depicted in Fig. 3 ▸ and mapped over the range −0.1763 to 1.2643 Å. Strong hydrogen-bond inter­actions, such as C—H⋯O, are seen as a bright-red area on the Hirshfeld surfaces (Şen *et al.*, 2017 ▸). The fingerprint plots over the Hirshfeld surfaces illustrate the significant differences between the inter­molecular inter­action patterns. In Fig. 4 ▸, it is observed N_inside_⋯H_outside_ = 2.3%, C_inside_⋯H_outside_ = 15.7%, O_inside_⋯H_outside_ = 29.7%, H_inside_⋯H_outside_ = 38% and all atoms_inside_⋯all atoms_outside_ = 100% of the total inter­actions. Fig. 4 ▸ shows that the major contributions are from H⋯H (38%) and O⋯H (30%) inter­actions. Fig. 5 ▸ illustrates the distribution of positive and negative potential over the Hirshfeld surfaces. Blue regions correspond to positive electrostatic potential (indicating hydrogen-bond donors) and the red regions to negative electrostatic potential (indicating hydrogen-bond acceptors) (Kumar *et al.*, 2013 ▸).

## IR spectroscopic analyses   

**Figure 6:**
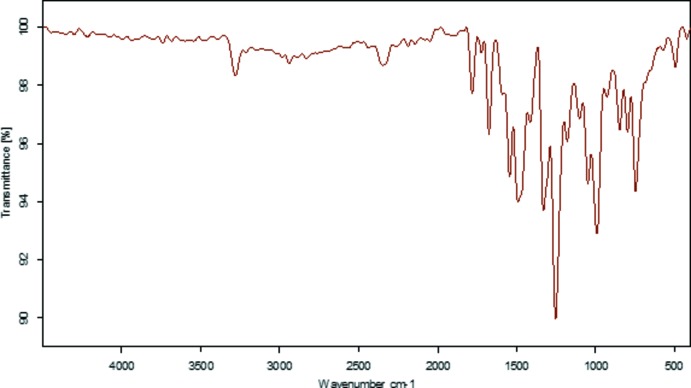
The FT–IR spectrum of the title compound.

The FT–IR spectrum of 2,3-dimeth­oxy-*N*-(4-nitro­phen­yl)benzamide, shown in Fig. 6 ▸, has several characterization bands. The first characteristic absorption band is at 3311 cm^−1^ and was assigned to the N—H stretching vibration. The second remarkable very strong vibrational band is located at 1689 cm^−1^ and can be attributed to the C=O stretching vibration. Another group wavenumber is the C—N stretching vibration that appears at 862 cm^−1^. This vibration frequency belongs to the nitro group attached to the phenyl ring at the 4-position. The asymmetrical and symmetrical stretching vibrations of the nitro group are observed at 1549 and 1327 cm^−1^, respectively. In the IR spectrum, peaks corresponding to –C=O– stretching and –NH– stretching indicate the presence of an amide linkage. These values are in agreement with those previously reported for similar compounds (Cakmak *et al.*, 2016 ▸; Demir *et al.*, 2015 ▸).

## Database survey   

A search of the Cambridge Structural Database (CSD, Version 5.38, last update May 2017; Groom *et al.*, 2016 ▸) for the 2,3-dimethyl-*N*-(phen­yl)benzamide skeleton gave 17 hits. One of these compounds, *viz*. 2,3-dimeth­oxy-*N*-(4-methyl­phen­yl)benzamide, also named as 2,3-dimeth­oxy-*N*-(*p*-tol­yl)benzamide (UYALEN; Cakmak *et al.*, 2016 ▸) is similar to the title compound. However, here the two aryl rings are inclined to one another by *ca* 34.16°, despite the presence of an intra­molecular N—H⋯O_meth­oxy_ hydrogen bond. A search for the 4-nitro­phenyl­benzamide skeleton gave 16 hits. They include 4-nitro­phenyl­benzamide itself, also called benz-*p*-nitro­anilide (BUTDID; Du Plessis *et al.*, 1983 ▸) and two polymorphs (ortho­rhom­bic and monoclinic) of 4′-nitro­salicylanilide (respectively, KADZEU and KADZIY; Etter *et al.*, 1988 ▸). Here, the aryl rings are inclined to one another by *ca* 62.30° in BUTDID, 11.24 (10)° in KADZEU, and 3.02 (12) and 2.69 (12)° in the two independent mol­ecules of the monoclinic polymorph of 4′-nitro­salicylanilide, *i.e.* KADZIY. The same dihedral angle in the title compound is 4.89 (9)°. Only in BUTDID, with a dihedral angle of *ca* 62.30°, is there no intra­molecular N—H⋯O hydrogen present.

## Synthesis and crystallization   

To a solution of 4-nitro­aniline (10 mmol) and tri­ethyl­amine (10 mmol) in THF (10 ml) was added dropwise a THF (10 ml) solution of 2,3-di­meth­oxy­benzoyl chloride (11 mmol) at room temperature. The reaction mixture was stirred at room temperature for 15 h and then the resulting white salt precipitate was filtered off and then 150 ml water was added dropwise to the filtrate. The precipitate was filtered off and washed several times with water to remove excessive aniline derivative and tri­methyl­amine hydro­chloride salt. The crude product was crystallized from aceto­nitrile (yield 2.09 g 63%; m.p. 448–451 K; Demir *et al.*, 2015 ▸; Cakmak *et al.*, 2016 ▸).

## Refinement   

Crystal data, data collection and structure refinement details are summarized in Table 2 ▸. The imino-H atom was located in a difference-Fourier map. All C-bound H atoms were positioned geometrically and refined using a riding model with C—H = 0.93–0.97 Å and *U*_iso_(H) = 1.2–1.5*U*_eq_(C).

## Supplementary Material

Crystal structure: contains datablock(s) I, global. DOI: 10.1107/S2056989017017741/xu5912sup1.cif

Structure factors: contains datablock(s) I. DOI: 10.1107/S2056989017017741/xu5912Isup2.hkl

Supporting information file. DOI: 10.1107/S2056989017017741/xu5912Isup3.cml

CCDC reference: 1580287

Additional supporting information:  crystallographic information; 3D view; checkCIF report

## Figures and Tables

**Table 1:** Hydrogen-bond geometry (Å, °)

*D*—H⋯*A*	*D*—H	H⋯*A*	*D*⋯*A*	*D*—H⋯*A*
N2—H2⋯O4	0.87 (2)	1.924 (19)	2.6805 (16)	144.6 (17)
C5—H5⋯O3^i^	0.93	2.48	3.2597 (19)	141

Symmetry code: (i) 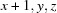.

**Table 2:** Experimental details

Crystal data	
Chemical formula	C_15_H_14_N_2_O_5_
*M*_r_	302.28
Crystal system, space group	Triclinic, *P* 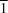
Temperature (K)	296
*a*, *b*, *c* (Å)	6.9293 (5), 7.3270 (5), 15.7411 (11)
α, β, γ (°)	94.198 (6), 96.189 (6), 116.053 (5)
*V* (Å^3^)	707.27 (9)
*Z*	2
Radiation type	Mo *K*α
μ (mm^−1^)	0.11
Crystal size (mm)	0.74 × 0.49 × 0.28

Data collection	
Diffractometer	Stoe IPDS 2
Absorption correction	Integration (*X-RED32*; Stoe & Cie, 2002 ▸)
*T*_min_, *T*_max_	0.947, 0.972
No. of measured, independent and observed [*I* > 2σ(*I*)] reflections	10204, 2776, 2011
*R*_int_	0.109
(sin θ/λ)_max_ (Å^−1^)	0.617

Refinement	
*R*[*F* ^2^ > 2σ(*F* ^2^)], *wR*(*F* ^2^), *S*	0.039, 0.114, 1.09
No. of reflections	2776
No. of parameters	203
H-atom treatment	H atoms treated by a mixture of independent and constrained refinement
Δρ_max_, Δρ_min_ (e Å^−3^)	0.16, −0.15

Computer programs: *X-AREA* and *X-RED32* (Stoe & Cie, 2002 ▸), *SHELXT* (Sheldrick, 2015*a* ▸), *SHELXL2017* (Sheldrick, 2015*b* ▸), *ORTEP-3 for Windows* (Farrugia, 2012 ▸), *WinGX* (Farrugia, 2012 ▸) and *PLATON* (Spek, 2009 ▸).

## supplementary crystallographic information

### Crystal data

**Table:**

C_15_H_14_N_2_O_5_	*Z* = 2
*M**_r_* = 302.28	*F*(000) = 316
Triclinic, *P*1	*D*_x_ = 1.419 Mg m^−^^3^
*a* = 6.9293 (5) Å	Mo *K*α radiation, λ = 0.71073 Å
*b* = 7.3270 (5) Å	Cell parameters from 12957 reflections
*c* = 15.7411 (11) Å	θ = 2.6–27.5°
α = 94.198 (6)°	µ = 0.11 mm^−^^1^
β = 96.189 (6)°	*T* = 296 K
γ = 116.053 (5)°	Prism, colorless
*V* = 707.27 (9) Å^3^	0.74 × 0.49 × 0.28 mm

### Data collection

**Table:**

Stoe IPDS 2 diffractometer	2776 independent reflections
Radiation source: sealed X-ray tube, 12 x 0.4 mm long-fine focus	2011 reflections with *I* > 2σ(*I*)
Plane graphite monochromator	*R*_int_ = 0.109
Detector resolution: 6.67 pixels mm^-1^	θ_max_ = 26.0°, θ_min_ = 2.6°
rotation method scans	*h* = −8→8
Absorption correction: integration (X-RED32; Stoe & Cie, 2002)	*k* = −9→9
*T*_min_ = 0.947, *T*_max_ = 0.972	*l* = −19→19
10204 measured reflections	

### Refinement

**Table:**

Refinement on *F*^2^	0 restraints
Least-squares matrix: full	Hydrogen site location: mixed
*R*[*F*^2^ > 2σ(*F*^2^)] = 0.039	H atoms treated by a mixture of independent and constrained refinement
*wR*(*F*^2^) = 0.114	*w* = 1/[σ^2^(*F*_o_^2^) + (0.0593*P*)^2^ + 0.0364*P*] where *P* = (*F*_o_^2^ + 2*F*_c_^2^)/3
*S* = 1.09	(Δ/σ)_max_ < 0.001
2776 reflections	Δρ_max_ = 0.16 e Å^−^^3^
203 parameters	Δρ_min_ = −0.15 e Å^−^^3^

### Special details

**Table:**

Geometry. All esds (except the esd in the dihedral angle between two l.s. planes) are estimated using the full covariance matrix. The cell esds are taken into account individually in the estimation of esds in distances, angles and torsion angles; correlations between esds in cell parameters are only used when they are defined by crystal symmetry. An approximate (isotropic) treatment of cell esds is used for estimating esds involving l.s. planes.

### Fractional atomic coordinates and isotropic or equivalent isotropic displacement parameters (Å^2^)

**Table:**

	*x*	*y*	*z*	*U*_iso_*/*U*_eq_	
O4	0.64070 (15)	0.29058 (17)	0.80665 (6)	0.0545 (3)	
O5	0.60933 (17)	0.26544 (19)	0.97197 (7)	0.0616 (3)	
N2	0.4382 (2)	0.2366 (2)	0.64515 (8)	0.0511 (3)	
O3	0.10391 (19)	0.2209 (2)	0.63299 (8)	0.0808 (4)	
C13	0.4424 (2)	0.2492 (2)	0.83248 (9)	0.0471 (3)	
C4	0.4797 (2)	0.2459 (2)	0.56018 (9)	0.0459 (3)	
C8	0.2622 (2)	0.2150 (2)	0.77272 (10)	0.0488 (4)	
O2	0.8200 (3)	0.2760 (3)	0.30063 (10)	0.1064 (5)	
N1	0.6417 (3)	0.2623 (2)	0.30988 (11)	0.0768 (5)	
C7	0.2601 (2)	0.2247 (2)	0.67740 (10)	0.0517 (4)	
C12	0.4247 (2)	0.2346 (2)	0.91990 (10)	0.0507 (4)	
C1	0.5861 (3)	0.2588 (2)	0.39713 (10)	0.0570 (4)	
C3	0.3267 (3)	0.2228 (3)	0.49000 (10)	0.0549 (4)	
H3	0.188086	0.202409	0.498155	0.066*	
O1	0.5080 (3)	0.2514 (3)	0.25036 (9)	0.1153 (6)	
C5	0.6860 (2)	0.2752 (2)	0.54688 (10)	0.0540 (4)	
H5	0.788868	0.291175	0.593672	0.065*	
C9	0.0660 (2)	0.1701 (3)	0.80232 (11)	0.0598 (4)	
H9	−0.055930	0.146485	0.763333	0.072*	
C6	0.7391 (3)	0.2808 (3)	0.46522 (11)	0.0597 (4)	
H6	0.876591	0.299130	0.456262	0.072*	
C11	0.2278 (3)	0.1919 (3)	0.94704 (10)	0.0610 (4)	
H11	0.215193	0.184597	1.005063	0.073*	
C2	0.3818 (3)	0.2305 (3)	0.40828 (10)	0.0594 (4)	
H2B	0.281017	0.216468	0.361048	0.071*	
C10	0.0500 (3)	0.1603 (3)	0.88803 (12)	0.0667 (5)	
H10	−0.081928	0.131915	0.906628	0.080*	
C14	0.6008 (3)	0.2573 (3)	1.06211 (10)	0.0672 (5)	
H14A	0.740231	0.281308	1.091532	0.101*	
H14B	0.563274	0.360514	1.084938	0.101*	
H14C	0.493006	0.124535	1.070342	0.101*	
C15	0.8027 (3)	0.5006 (3)	0.83037 (13)	0.0752 (5)	
H15A	0.935274	0.518241	0.810544	0.113*	
H15B	0.751827	0.589244	0.804407	0.113*	
H15C	0.828921	0.534368	0.891963	0.113*	
H2	0.540 (3)	0.244 (3)	0.6846 (12)	0.072 (5)*	

### Atomic displacement parameters (Å^2^)

**Table:**

	*U*^11^	*U*^22^	*U*^33^	*U*^12^	*U*^13^	*U*^23^
O4	0.0428 (5)	0.0755 (8)	0.0474 (6)	0.0287 (5)	0.0087 (4)	0.0056 (5)
O5	0.0594 (6)	0.0816 (8)	0.0443 (6)	0.0319 (6)	0.0082 (5)	0.0108 (5)
N2	0.0453 (7)	0.0696 (9)	0.0424 (7)	0.0301 (6)	0.0038 (5)	0.0075 (6)
O3	0.0560 (7)	0.1396 (13)	0.0604 (7)	0.0554 (8)	0.0078 (5)	0.0211 (7)
C13	0.0438 (7)	0.0507 (9)	0.0508 (8)	0.0236 (6)	0.0127 (6)	0.0076 (6)
C4	0.0485 (8)	0.0452 (8)	0.0443 (7)	0.0217 (6)	0.0051 (6)	0.0057 (6)
C8	0.0440 (7)	0.0521 (9)	0.0522 (8)	0.0228 (6)	0.0102 (6)	0.0078 (7)
O2	0.1207 (13)	0.1259 (14)	0.0872 (11)	0.0572 (11)	0.0587 (10)	0.0277 (9)
N1	0.1039 (13)	0.0702 (10)	0.0590 (9)	0.0362 (9)	0.0319 (9)	0.0164 (7)
C7	0.0443 (8)	0.0595 (10)	0.0522 (8)	0.0245 (7)	0.0057 (6)	0.0076 (7)
C12	0.0531 (8)	0.0515 (9)	0.0494 (8)	0.0245 (7)	0.0104 (6)	0.0094 (7)
C1	0.0736 (10)	0.0493 (9)	0.0486 (8)	0.0258 (8)	0.0177 (7)	0.0102 (7)
C3	0.0518 (8)	0.0631 (10)	0.0505 (9)	0.0272 (7)	0.0043 (6)	0.0090 (7)
O1	0.1461 (15)	0.1591 (17)	0.0487 (8)	0.0731 (13)	0.0207 (9)	0.0256 (9)
C5	0.0496 (8)	0.0629 (10)	0.0515 (8)	0.0278 (7)	0.0061 (6)	0.0063 (7)
C9	0.0451 (8)	0.0729 (11)	0.0647 (10)	0.0278 (8)	0.0127 (7)	0.0157 (8)
C6	0.0586 (9)	0.0623 (11)	0.0634 (10)	0.0293 (8)	0.0201 (8)	0.0107 (8)
C11	0.0670 (10)	0.0690 (11)	0.0549 (9)	0.0325 (8)	0.0256 (8)	0.0196 (8)
C2	0.0682 (10)	0.0594 (10)	0.0472 (8)	0.0273 (8)	0.0010 (7)	0.0092 (7)
C10	0.0523 (9)	0.0820 (13)	0.0743 (11)	0.0318 (9)	0.0277 (8)	0.0247 (9)
C14	0.0816 (12)	0.0716 (12)	0.0448 (9)	0.0317 (9)	0.0075 (8)	0.0086 (8)
C15	0.0497 (9)	0.0840 (14)	0.0778 (12)	0.0158 (9)	0.0176 (8)	0.0110 (10)

### Geometric parameters (Å, º)

**Table:**

O4—C13	1.3849 (16)	C1—C6	1.371 (2)
O4—C15	1.441 (2)	C3—C2	1.379 (2)
O5—C12	1.3612 (18)	C3—H3	0.9300
O5—C14	1.4311 (19)	C5—C6	1.373 (2)
N2—C7	1.3547 (19)	C5—H5	0.9300
N2—C4	1.3983 (19)	C9—C10	1.370 (2)
N2—H2	0.870 (19)	C9—H9	0.9300
O3—C7	1.2115 (18)	C6—H6	0.9300
C13—C8	1.394 (2)	C11—C10	1.380 (2)
C13—C12	1.402 (2)	C11—H11	0.9300
C4—C3	1.391 (2)	C2—H2B	0.9300
C4—C5	1.392 (2)	C10—H10	0.9300
C8—C9	1.393 (2)	C14—H14A	0.9600
C8—C7	1.506 (2)	C14—H14B	0.9600
O2—N1	1.221 (2)	C14—H14C	0.9600
N1—O1	1.216 (2)	C15—H15A	0.9600
N1—C1	1.465 (2)	C15—H15B	0.9600
C12—C11	1.383 (2)	C15—H15C	0.9600
C1—C2	1.370 (2)		
			
C13—O4—C15	113.96 (12)	C6—C5—H5	119.7
C12—O5—C14	117.60 (13)	C4—C5—H5	119.7
C7—N2—C4	129.19 (13)	C10—C9—C8	120.98 (15)
C7—N2—H2	113.0 (12)	C10—C9—H9	119.5
C4—N2—H2	117.7 (12)	C8—C9—H9	119.5
O4—C13—C8	120.97 (12)	C1—C6—C5	118.91 (15)
O4—C13—C12	118.37 (12)	C1—C6—H6	120.5
C8—C13—C12	120.61 (13)	C5—C6—H6	120.5
C3—C4—C5	119.50 (14)	C10—C11—C12	119.99 (14)
C3—C4—N2	123.89 (13)	C10—C11—H11	120.0
C5—C4—N2	116.58 (13)	C12—C11—H11	120.0
C9—C8—C13	118.36 (14)	C1—C2—C3	119.54 (15)
C9—C8—C7	116.01 (13)	C1—C2—H2B	120.2
C13—C8—C7	125.63 (13)	C3—C2—H2B	120.2
O1—N1—O2	123.34 (17)	C9—C10—C11	120.66 (14)
O1—N1—C1	118.39 (18)	C9—C10—H10	119.7
O2—N1—C1	118.27 (18)	C11—C10—H10	119.7
O3—C7—N2	122.79 (14)	O5—C14—H14A	109.5
O3—C7—C8	120.31 (13)	O5—C14—H14B	109.5
N2—C7—C8	116.90 (12)	H14A—C14—H14B	109.5
O5—C12—C11	125.07 (14)	O5—C14—H14C	109.5
O5—C12—C13	115.56 (13)	H14A—C14—H14C	109.5
C11—C12—C13	119.37 (14)	H14B—C14—H14C	109.5
C2—C1—C6	121.87 (15)	O4—C15—H15A	109.5
C2—C1—N1	119.17 (16)	O4—C15—H15B	109.5
C6—C1—N1	118.95 (16)	H15A—C15—H15B	109.5
C2—C3—C4	119.64 (15)	O4—C15—H15C	109.5
C2—C3—H3	120.2	H15A—C15—H15C	109.5
C4—C3—H3	120.2	H15B—C15—H15C	109.5
C6—C5—C4	120.53 (14)		
			
C15—O4—C13—C8	107.52 (16)	O1—N1—C1—C2	4.1 (3)
C15—O4—C13—C12	−74.91 (17)	O2—N1—C1—C2	−175.90 (16)
C7—N2—C4—C3	−6.9 (3)	O1—N1—C1—C6	−176.93 (17)
C7—N2—C4—C5	174.65 (15)	O2—N1—C1—C6	3.1 (2)
O4—C13—C8—C9	178.74 (14)	C5—C4—C3—C2	−0.4 (2)
C12—C13—C8—C9	1.2 (2)	N2—C4—C3—C2	−178.75 (15)
O4—C13—C8—C7	−2.0 (2)	C3—C4—C5—C6	−0.2 (2)
C12—C13—C8—C7	−179.54 (15)	N2—C4—C5—C6	178.27 (14)
C4—N2—C7—O3	−0.2 (3)	C13—C8—C9—C10	0.2 (2)
C4—N2—C7—C8	179.49 (14)	C7—C8—C9—C10	−179.11 (16)
C9—C8—C7—O3	9.6 (2)	C2—C1—C6—C5	−0.3 (3)
C13—C8—C7—O3	−169.65 (16)	N1—C1—C6—C5	−179.30 (15)
C9—C8—C7—N2	−170.15 (14)	C4—C5—C6—C1	0.6 (2)
C13—C8—C7—N2	10.6 (2)	O5—C12—C11—C10	−178.99 (15)
C14—O5—C12—C11	−1.3 (2)	C13—C12—C11—C10	1.3 (2)
C14—O5—C12—C13	178.43 (14)	C6—C1—C2—C3	−0.2 (3)
O4—C13—C12—O5	0.7 (2)	N1—C1—C2—C3	178.71 (15)
C8—C13—C12—O5	178.26 (14)	C4—C3—C2—C1	0.6 (2)
O4—C13—C12—C11	−179.54 (14)	C8—C9—C10—C11	−0.9 (3)
C8—C13—C12—C11	−2.0 (2)	C12—C11—C10—C9	0.1 (3)

### Hydrogen-bond geometry (Å, º)

**Table:**

*D*—H···*A*	*D*—H	H···*A*	*D*···*A*	*D*—H···*A*
N2—H2···O4	0.870 (19)	1.924 (19)	2.6805 (16)	144.6 (17)
C5—H5···O3^i^	0.93	2.48	3.2597 (19)	141

Symmetry code: (i) *x*+1, *y*, *z*.
